# University of Buffalo

**Published:** 1857-03

**Authors:** 


*University of Buffalo.— Medical Department.*—We publish elsewhere, a report of the commencement ceremonies of Buffalo Medical College. The session just closed has been a pleasant and harmonious one. No interruptions of any kind have occurred, and we have no recollection of a term during which the students were so thoroughly worked.

At a meeting of the Faculty, held just before the close of the session, Dr. Austin W. Nichols was appointed Demonstrator of Anatomy, *vice* Dr. Chas. Ap A. Bowen, recently appointed Professor of Anatomy, at Toronto. Resolutions were also passed thanking Dr. Nichols for his otherwise unrewarded services during the winter as Clinical Assistant, and as proxy for the Demonstrator. Prevented from attending the meeting ourselves, we here join cordially in the resolution. Dr. N. has given evidence of high capacity and persevering industry, and we congratulate the future students of the school on the appointment of an excellent practical teacher.

---

*University of Buffalo.— Summer Course.*—We call the especial attention of preceptors and students to the establishment of a summer course in this institution. The intention is to afford the highest advantages attainable. Students are apt to study a close economy in selecting their residences. It should be borne in mind that in the brief term of three years allotted to preparation to medical students in this country, it is very essential that that time should be well employed. The curriculum provided by this summer course is so arranged as to afford a constant stimulus to thorough and profitable reading. The intermingling of lectures with familiar recitations, will afford a pleasant variety, and give abundant opportunity for enlightenment on obscure points.

The expense attending this course is merely nominal—the additional cost of residence in a city being the only expense occurred. As a summer residence, Buffalo has no equal in the United States, for a delicious coolness and salubrity. For particulars we refer the reader to third page of cover.

